# Utility of a penile compression device for the quality of life in male patients with urinary incontinence after prostatectomy (the MORE study): a randomized prospective study

**DOI:** 10.1186/s13104-023-06564-z

**Published:** 2023-10-18

**Authors:** Daisuke Gotoh, Kazumasa Torimoto, Kenta Onishi, Yosuke Morizawa, Shunta Hori, Yasushi Nakai, Makito Miyake, Kiyohide Fujimoto

**Affiliations:** Department of Urology, Nara Medical University, 840, Shijo-cho, Kashihara, Nara Japan

**Keywords:** Urinary incontinence, Radical prostatectomy, Quality of life

## Abstract

**Objectives:**

To verify the effects of penile clamping on the degree of stress urinary incontinence and quality of life in post-radical prostatectomy patients.

**Results:**

Thirty-seven patients suffering from stress urinary incontinence after undergoing radical prostatectomy were enrolled. A total of 19 and 18 patients were analyzed in the non-clamp and clamp groups, respectively. The mean ages of the patients in non-clamp and clamp groups were 68.3 ± 7.1 years and 71.2 ± 4.8 years, respectively; the mean time after radical prostatectomy was 28.9 ± 44.0 months and 26.2 ± 39.0 months, respectively. The penile clamp used was the CLAMPMED® (URINE CONTROL CLAMP) size M (MURANAKA MEDICAL INSTRUMENTS Co., Ltd.). Specific urinary care pads (Sawayaka Pad for Men, Small Quantity®, Unicharm Corporation), were provided; the average daily usage was monitored for four weeks. The quality of life was evaluated using the King’s Health Questionnaire. The average daily use of urinary care pads was significantly reduced in the clamp group than in the non-clamp group (-0.83 ± 1.51 vs. -0.16 ± 0.69, P = 0.0071). King’s Health Questionnaire scores did not change significantly in either group. Wearing the CLAMPMED® reduced the amount of urinary incontinence but did not improve the quality of life.

**Trial registration:**

The Japan Registry of Clinical Trials (jRCT1052230083). Registered 2 August, 2023.

## Introduction

In Japan, more than 20,000 patients undergo radical prostatectomies annually for prostate cancer. Severe urinary incontinence is observed in most patients immediately after surgery, but in more than 90% of patients, it becomes mild or less severe within 6 months to 1 year after surgery. However, 1–2% have prolonged severe urinary incontinence and impaired quality of life (QOL) [1]. These patients manage urinary incontinence with urine pads or diapers, and some patients undergo artificial urethral sphincter implantation in severe cases more than a year after surgery. Urinary pads and diapers cannot be said to be comfortable, even though they perform well. In addition, although an artificial urinary sphincter significantly improves QOL [2], it is not a convenient measure against urinary incontinence because it requires surgery and carries the risk of complications such as infection. Male urinary incontinence prevention medical devices exist but are not widely used in Japan because their utility is not well known to patients or medical professionals and there is little evidence that these are useful for urinary incontinence. We believe that using a urinary incontinence prevention device can be a simple measure against urinary incontinence for patients who are recovering from urinary incontinence after radical prostatectomy or are still affected by urinary incontinence more than one year after surgery. Therefore, in this study, we investigated whether the use of clamps for incontinence after surgery would reduce the amount of incontinence and improve the QOL in patients affected by stress urinary incontinence after radical prostatectomy.

## Methods

**Fig. 1:**
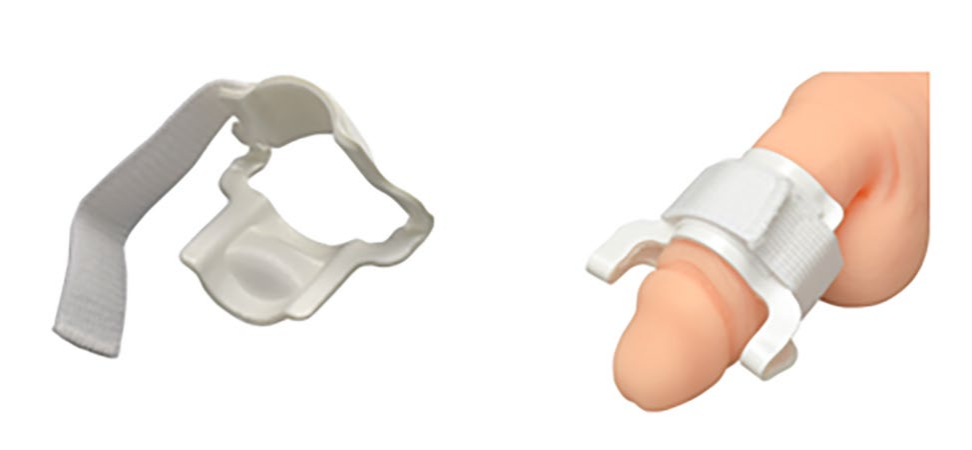
Diagram of CLAMPMED® used. We reprinted this figure from the website of Muranaka Medical Instruments Co., Ltd

This was a prospective, randomized, and interventional study. Between January 1, 2018, and March 31, 2019, the participants were patients who visited Nara Medical University Hospital and were affected by stress urinary incontinence after undergoing radical prostatectomy. The exclusion criteria were as follows: patients who have undergone radical prostatectomy within the last month, patients with severe penile skin disease, patients with artificial urethral sphincters, and patients with psychosis or psychiatric symptoms who had difficulty participating in the study. The penile clamp used was the CLAMPMED® (URINE CONTROL CLAMP) size M (MURANAKA MEDICAL INSTRUMENTS Co., Ltd.) (Fig. 1). Patients used the clamp only during the day and undertook 15-minute breaks every 2–3 h. After obtaining informed consent to participate in the study, we provided a specific urinary care 20 mL absorbent pad (Sawayaka Pad for Men, Small Quantity®, Unicharm corporation), checked the average daily usage for two weeks, and evaluated each participant using the King’s Health Questionnaire (KHQ). Subsequently, the participants were randomly assigned to the clamp-use or non-clamp-use groups. After 4 weeks, the average daily usage of urinary care pads was monitored, and the KHQ evaluation was performed again.

The KHQ was designed to evaluate the global and specific impacts of urinary incontinence on QOL. It has established validity and reliability and is available in several languages [3]. It addresses nine different domains in 21 items with Likert-scale response options (range 1–4 and 1–5 for the first option). The domains were general health perception, the impact of incontinence, role limitations, physical and social limitations, personal relationships, emotions, sleep/energy, and the severity of coping measures. An additional independent scale with nine questions was designed to evaluate symptom severity perception [4]. Scores for each domain were calculated using a complex system that manages missing values and results, ranging from 0 to 100, with higher scores indicating a more impaired QOL. The KHQ was administered at baseline and follow-up visits. We used the global score calculated from the sum of the crude results obtained for each item (range: 18–85 points) and missing values, which were allowed in the personal relationship domain, were scored as zero.

In this study, MURANAKA MEDICAL INSTRUMENTS CO. Ltd. provided penile clamps and urinary care pads.

### Statistical analysis

All values are expressed as mean ± standard deviation. The Mann–Whitney U test and Wilcoxon matched-pairs test were used to evaluate statistical differences. Prism software ver. 8.4.2 (GraphPad Software, San Diego, CA, USA) was used for the statistical analyses and data plotting. Statistical significance was set at P < 0.05.

## Results

**Fig. 2:**
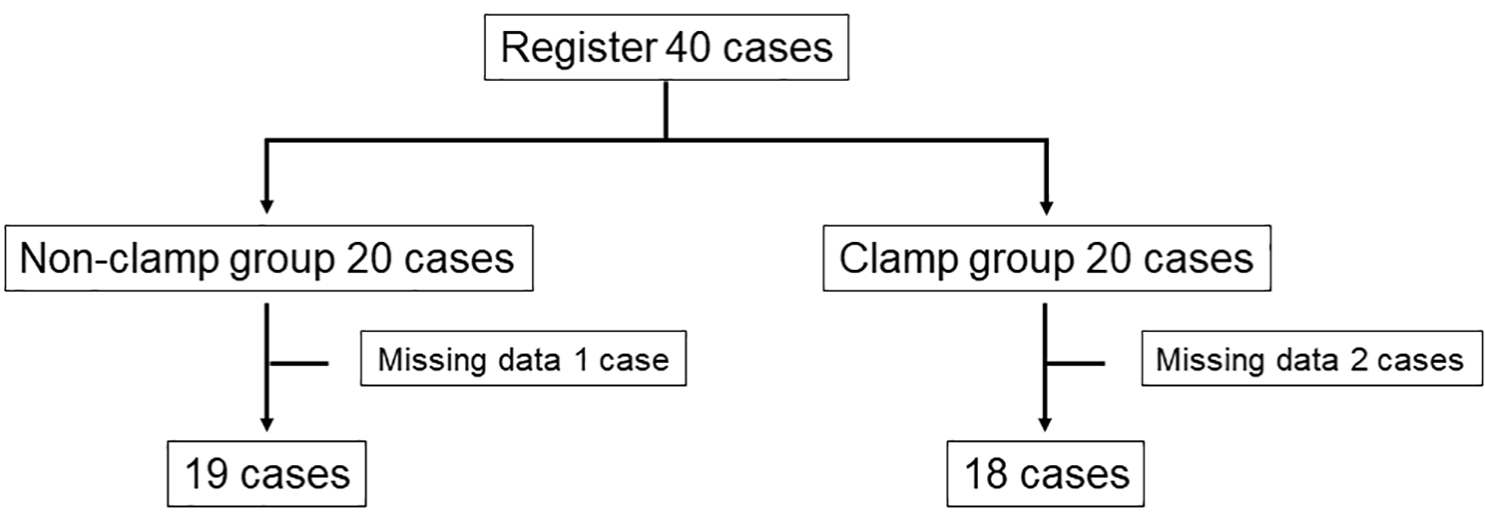
Research flow. Although 40 patients were enrolled, only 19 and 18 patients in the non-clamp and clamp groups, respectively, were analyzed because of a lack of data

**Fig. 3:**
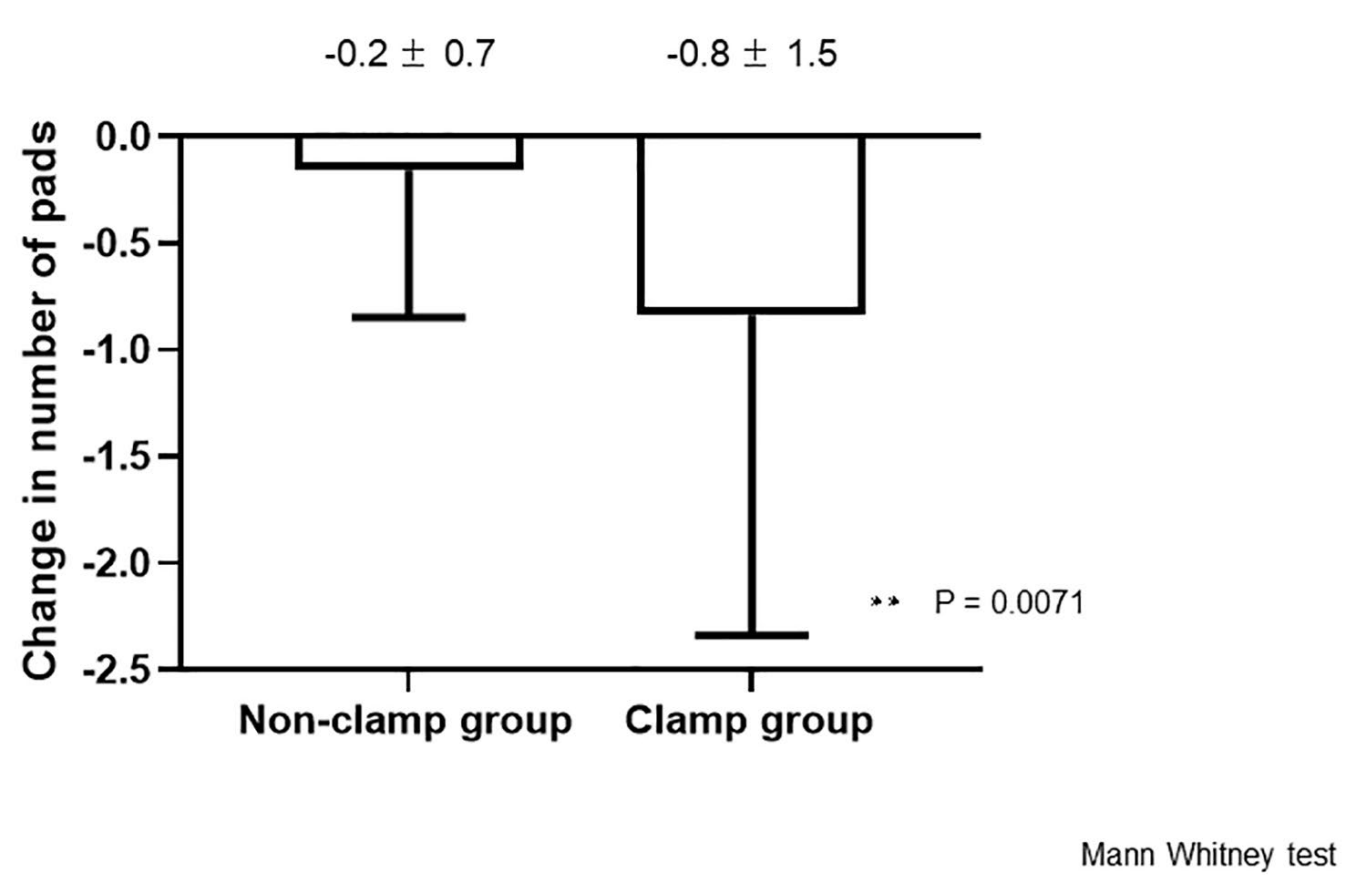
Changes in number of pads. The number of pads was significantly reduced in the clamp group than in the non-clamp group (-0.83 ± 1.51 vs. -0.16 ± 0.69, P = 0.0071)

Although 40 patients were enrolled, only 19 and 18 patients in the non-clamp and clamp groups, respectively, were analyzed because of a lack of data (Fig. 2). The mean ages of the patients in non-clamp and clamp groups were 68.3 ± 7.1 years and 71.2 ± 4.8 years, respectively. The mean periods after radical prostatectomy were 28.9 ± 44.0 months for the non-clamp group and 26.2 ± 39.0 months for the clamp group. In the clamp group, 15 patients underwent robot-assisted radical prostatectomy (RARP) and 3 underwent open retropubic radical prostatectomy (RRP); in the non-clamp group, 15 patients underwent RARP and 4 underwent open RRP. The mean baseline daily usages of urinary care pads of the non-clamp group and clamp group were 5.0 ± 3.4 and 5.7 ± 2.8 sheets, respectively (Table 1). However, the number of pads was significantly reduced in the clamp group compared to that in the non-clamp group (-0.83 ± 1.51 vs. -0.16 ± 0.69, P = 0.0071) (Fig. 3). The KHQ scores did not significantly change in either group (Table 2). Some patients complained of penile pain and irritation of the surrounding area due to clamping; however, both symptoms were mild.

**Table 1:** Patient characteristics

	Non-clamp group	Clamp group	P value
Patients (cases)	19	18	
Age (years)	68.3 ± 7.1	71.2 ± 4.8	0.3454
Period after radical prostatectomy (months)	28.9 ± 44.0	26.2 ± 39.0	0.6120
Pads used per day (sheets)	5.0 ± 3.4	5.7 ± 2.8	0.2901

Mann–Whitney U test

**Table 2:** Results of the King’s Health Questionnaire

	Non-clamp group		P value	Clamp group		P value
	Before	After		Before	After	
General health perception	27.6 ± 20.2	36.8 ± 22.6	0.1250	31.9 ± 24.0	27.8 ± 20.8	0.5625
Incontinence impact	59.6 ± 30.6	57.9 ± 29.1	0.5977	66.7 ± 25.6	63.0 ± 27.7	0.6250
Role limitations	45.6 ± 23.5	43.0 ± 21.7	0.2969	53.7 ± 31.1	54.6 ± 26.7	> 0.9999
Physical limitations	49.1 ± 25.7	46.5 ± 23.9	0.6719	61.1 ± 26.2	58.3 ± 30.3	0.4209
Social limitations	31.6 ± 21.0	31.6 ± 22.6	0.8750	44.4 ± 23.5	40.7 ± 27.5	0.4542
Personal relationship	23.5 ± 27.7	14.6 ± 22.7	0.1250	28.7 ± 37.4	35.2 ± 42.0	0.3848
Emotions	45.0 ± 26.3	46.2 ± 27.5	0.7813	51.2 ± 26.4	51.2 ± 30.0	> 0.9999
Sleep and energy	41.2 ± 30.1	35.1 ± 23.5	0.3438	41.7 ± 28.2	44.4 ± 29.2	0.4902
Severity coping measures	53.3 ± 23.7	51.6 ± 23.2	0.9844	57.4 ± 19.7	57.0 ± 21.8	0.9323

Wilcoxon matched-pairs signed rank test

## Discussion

Radical prostatectomy is widely performed to treat localized prostate cancer. Radical prostatectomy is a surgical procedure in which the prostate and seminal vesicles are cut from the bladder neck and urethra and removed as a lump, and the cut bladder neck and urethra are anastomosed. Damage to the sphincter is inevitable during urethral transection. The symptoms of stress urinary incontinence are most severe immediately after surgery and tend to improve over time. It is difficult to expect further improvement after one year postoperatively, and severe urinary incontinence after radical prostatectomy is thought to occur in approximately 1% of cases. The incidence of stress incontinence after laparoscopic RARP, which has become widespread in recent years, is similar to that after RRP [5]. Pelvic floor muscle training, which is a type of physical therapy, accelerates the recovery of urinary incontinence after radical prostatectomy but is said to be ineffective after 1 year postoperatively [6].

In Japan, the awareness and usage of incontinence surgical clamps used for male stress urinary incontinence are extremely low, and the only therapeutic options are to undergo artificial urethral sphincter implantation and incontinence prevention surgery with unstable results or to use urine pads and diapers. For patients who are recovering from urinary continence after radical prostatectomy or who still suffer from urinary incontinence more than 1 year after surgery, incontinence surgical clamps could be a new therapeutic option.

There are few reports of penile clamps in Japan; however, some cases have been reported overseas. A Canadian study compared the effectiveness of three types of penile clamps. The CLAMPMED® isoform significantly reduced incontinence in the 4-hour pad test (122.8 ± 130.8 vs. 32.3 ± 24.3 g) [7]. A 2015 New Zealand study on the usefulness of surgical incontinence clamps investigated the effect of surgical incontinence clamps (Dribblestop®) on urinary incontinence in 16 patients who had undergone radical prostatectomy. The incontinence impact questionnaire (IIQ-7) was used for the evaluation. The IIQ-7 score decreased significantly (P < 0.001) from 67.3 (range 28.6–95.2) before treatment to 26.8 (range 0–66.7) after treatment [8]. A 2016 Israeli study on a penile clamp device identified the design characteristics, including envelopment, adaptability, and durability, that provide the safest mechanical conditions in the penis and thus minimize the risk of tissue damage while still managing incontinence [9].

However, in this study, CLAMPMED® reduced the number of pads but did not improve the QOL. The average Japanese penis size is 7.9 ± 2.0 cm around 70 years old, while the overseas average is around 15 cm [10, 11]. Although it is not possible to make a simple comparison, we believe that overseas markets are more accessible than those in Japan. In this study, it might be thought that there were many cases in which it was not possible to wear the device stably because the penises of the research participants were smaller than those recommended for use with the CLAMPMED®. In Japan, the existence of penis clamps is not widely recognized, however we urologists are aware of the existence of penile clamps, so it is important to educate patients and other medical professionals about the existence of penile clamps.

### Limitations

This study had some limitations. First, the sample size was too small to draw. definitive conclusions. Thus, further studies with a larger number of participants are required to confirm our findings. Second, we did not use a clamping device developed in Japan. In hindsight, a device developed in Japan and adapted to the size of the Japanese population should have been used. Third, we did not perform urodynamic studies or storage function tests using a clamp device on any patient. Fourth, the evaluation of discomfort and pain is important for penile clamp devices because the structure of the device may cause damage to the skin tissue and adipose tissue [7, 9]. Therefore, a future study involving the evaluation of penile clamp device-related discomfort and pain is necessary.

## Conclusion

Although wearing the CLAMPMED® reduced the amount of urinary incontinence, it did not improve quality of life. The effect could have been better if the size of the CLAMPMED® and the penis were appropriate.

## Data Availability

The datasets used and/or analyzed during the current study are available from the corresponding author on reasonable request.

## Abbreviations

KHQ: King’s Health Questionnaire
QOL: Quality of life
RARP: Robot-assisted radical prostatectomy
RRP: Retropubic radical prostatectomy

## References

[1] Bauer RM, Bastian PJ, Gozzi C, Stief CG (2009). Postprostatectomy incontinence: all about diagnosis and management. Eur Urol.

[2] Van der Aa F, Drake MJ, Kasyan GR, Petrolekas A, Cornu JN, Young Academic Urologists Functional Urology Group (2013). The artificial urinary sphincter after a quarter of a century: a critical systematic review of its use in male non-neurogenic incontinence. Eur Urol.

[3] Viana R, Viana S, Neto F, Mascarenhas T (2015). Adaptation and validation of the King’s Health Questionnaire in Portuguese women with urinary incontinence. Int Urogynecol J.

[4] Kelleher CJ, Cardozo LD, Khullar V, Salvatore S (1997). A new questionnaire to assess the quality of life of urinary incontinent women. Br J Obstet Gynaecol.

[5] Haglind E, Carlsson S, Stranne J, Wallerstedt A, Wilderäng U, Thorsteinsdottir T (2015). Urinary incontinence and erectile dysfunction after robotic *versus* open radical prostatectomy: a prospective, controlled, nonrandomised trial. Eur Urol.

[6] Filocamo MT, Li Marzi V, Del Popolo G, Cecconi F, Marzocco M, Tosto A (2005). Effectiveness of early pelvic floor rehabilitation treatment for post-prostatectomy incontinence. Eur Urol.

[7] Moore KN, Schieman S, Ackerman T, Dzus HY, Metcalfe JB, Voaklander DC (2004). Assessing comfort, safety, and patient satisfaction with three commonly used penile compression devices. Urology.

[8] Barnard J, Westenberg AM (2015). The penile clamp: medieval pain or makeshift gain?. Neurourol Urodyn.

[9] Levy A, Fader M, Bader D, Gefen A (2017). Penile compression clamps: a model of the internal mechanical state of penile soft tissues. Neurourol Urodyn.

[10] Suzuki M, Ikegaya H, Idota N, Kawai T, Sato Y, Kume H (2019). Penile size and stretched rate in a Japanese male population: a cross-sectional cadaveric study. Int J Urol.

[11] Prause N, Park J, Leung S, Miller G (2015). Women’s preferences for penis size: a new research method using selection among 3D models. PLoS ONE.

