# Correction: Utility of a penile compression device for the quality of life in male patients with urinary incontinence after prostatectomy (the MORE study): a randomized prospective study

**DOI:** 10.1186/s13104-024-06732-9

**Published:** 2024-03-12

**Authors:** Daisuke Gotoh, Kazumasa Torimoto, Kenta Onishi, Yosuke Morizawa, Shunta Hori, Yasushi Nakai, Makito Miyake, Kiyohide Fujimoto

**Affiliations:** Department of Urology, Nara Medical University, 840, Shijo-cho, Kashihara, Nara Japan

**Correction: BMC Research Notes (2023) 16:277**

10.1186/s13104-023-06564-z

Following the publication of the original article, we were informed that co-author Kiyohide Fujimoto’s first and last names were inadvertently interchanged.

The correct name is shown in the author list of this Correction. The original article has been revised.

